# *Pseudomonas fluorescens* Pneumonia: A Case Report and Review of the Literature

**DOI:** 10.3390/microorganisms14061169

**Published:** 2026-05-22

**Authors:** Kadir Burak Akgün

**Affiliations:** Department of Pulmonology, Hatay Mustafa Kemal University, 31060 Hatay, Türkiye; kadirburak.akgun@mku.edu.tr; Tel.: +90-5448127828

**Keywords:** opportunistic infections, pneumonia, *Pseudomonas fluorescens*

## Abstract

*Pseudomonas fluorescens* is a rare, environmental Gram-negative bacterium that has been rarely reported as a cause of respiratory tract infections. This paper presents a case of a 72-year-old male who developed community-acquired pneumonia due to *P. fluorescens*. The diagnosis was made by sputum culture and he responded to meropenem treatment. A literature search revealed three previously reported cases of *P. fluorescens* pneumonia. These cases primarily affected elderly male patients. All reported patients demonstrated positive clinical outcomes following appropriate antimicrobial therapy. This case highlights that although *P. fluorescens* is often considered a colonizer, it may act as a potential pathogen in selected clinical settings.

## 1. Introduction

*Pseudomonas fluorescens* (*P. fluorescens*) is a diverse Gram-negative bacterium commonly found in soil and water [1]. Despite its established environmental role, its clinical significance in humans remains uncertain. Traditionally, it has not been considered a respiratory pathogen, and its presence in clinical specimens has often been attributed to colonization or contamination [2]. However, recent case reports and studies suggest that *P. fluorescens* may act as a true opportunistic pathogen under favorable host and environmental conditions. In the literature, *P. fluorescens* has been documented as a potential pathogen in various clinical presentations, including acute bronchitis and drowning-associated pneumonia [3,4]. Culture-independent molecular studies have shown that *P. fluorescens* can be detected as the predominant species in bronchoalveolar lavage fluids of symptomatic patients, even when standard cultures remain negative, indicating that its prevalence in respiratory infections may be underestimated [5]. Furthermore, its inclusion alongside major respiratory pathogens in recently developed multiplex PCR diagnostic panels underscores its increasing recognition as a clinically significant isolate [6].

This report presents a rare case of *P. fluorescens* pneumonia, highlighting the critical importance of diagnostic rigor in identifying this opportunistic pathogen. Unlike previous reports, we focus on the necessity of repeated isolation and strict microscopic quality criteria to confirm clinical significance, while providing a structured literature comparison to enhance the understanding of this rare clinical entity.

## 2. Case Presentation

A 72-year-old male patient with a prior diagnosis of chronic obstructive pulmonary disease (COPD) and previous treatment for prostate adenocarcinoma presented with progressively worsening cough, sputum production, and shortness of breath for two months. The sputum was green and sticky, and the shortness of breath was exertional and partially resolved at rest. There was no chest pain, hemoptysis, chills, or shivering; he complained of occasional fever. The patient reported an unintentional weight loss of approximately 4–5 kg over three months. His symptoms persisted despite treatment with several oral antibiotics, including amoxicillin/clavulanate, clarithromycin, moxifloxacin, and levofloxacin. He was a former smoker with a 30 pack-year history, who had quit smoking 20 years prior.

On physical examination, vital signs were within the normal range. Chest auscultation revealed bronchovesicular sounds and fine rales, particularly in the upper lung regions. Chest radiography showed increased reticulonodular opacities, more pronounced in the upper and middle lung areas. High-resolution computed tomography (HRCT) of the chest revealed underlying bilateral bronchiectasis with superimposed acute inflammatory changes, notably bronchial wall thickening and prominent tree-in-bud opacities. These findings strongly suggest an active bacterial or mycobacterial infection, lacking any specific hallmarks of invasive fungal disease (e.g., cavitation or halo sign) (Figure 1). Metastatic disease was suspected due to the oncological history, but gallium scintigraphy did not show malignant uptake, supporting the conclusion of an infection.

At the initial visit, a routine sputum culture and three consecutive acid-fast bacilli (AFB) samples were obtained. The patient was monitored without empirical antibiotic therapy. While all AFB smears and mycobacterial cultures—performed using both the BD BACTEC™ MGIT™ 960 liquid culture system and Löwenstein-Jensen (LJ) solid medium—showed no typical or nontuberculosis mycobacteria (NTM) growth, the routine culture yielded *P. fluorescens*. This isolate was identified utilizing the VITEK^®^ 2 Compact automated system (bioMérieux, Marcy-L’etoile, France). Minimum inhibitory concentration (MIC) values were interpreted in accordance with the European Committee on Antimicrobial Susceptibility Testing (EUCAST) breakpoints.

Upon the patient’s return for the control visit, and following a multidisciplinary consultation with the microbiology department, a second sputum sample was immediately obtained. To strictly rule out oropharyngeal contamination, the microscopic quality of the second sputum sample was evaluated before culturing. Direct Gram stain microscopy revealed <10 squamous epithelial cells and >25 polymorphonuclear leukocytes per low-power field (×100), confirming a high-quality lower respiratory tract specimen according to the Murray–Washington criteria. At higher magnification (×1000), abundant leukocytes and Gram-negative bacilli were observed.

Based on these definitive Gram stain findings, the initial culture result, and the patient’s clinical and radiological status, he was hospitalized. Intravenous meropenem was initiated according to the susceptibility profile of the first isolate. Antimicrobial susceptibility testing was performed, and the detailed Minimum Inhibitory Concentration (MIC) values are summarized in Table 1. The isolate’s intermediate susceptibility or resistance to several drug classes suggests that the patient’s extensive prior exposure to broad-spectrum oral antibiotics likely exerted a selective pressure, facilitating the colonization and subsequent emergence of this opportunistic pathogen. During the hospital stay, the second sputum culture also confirmed the growth of *P. fluorescens*.

On admission, the white blood cell (WBC) count was 8010/μL, with a neutrophil predominance of 77.3%. The patient demonstrated rapid clinical improvement; by the second day of targeted treatment, his cough, sputum production, and shortness of breath had significantly alleviated. Inflammatory markers trended down consistently throughout the treatment course. By day 3, the WBC count was 7820/μL (73.3% neutrophils), and it decreased to 3940/μL (56.6% neutrophils) by day 7. C-reactive protein (CRP) levels showed a concordant downward trend, decreasing from a baseline of 80 mg/L to 22 mg/L on day 3, and finally to 4 mg/L on day 7. Given the patient’s advanced age, underlying severe COPD, and this rapid clinical and biochemical response to targeted therapy, an invasive bronchoscopic sampling (such as bronchoalveolar lavage) was deemed clinically unnecessary and avoided to minimize procedural risks. The meropenem therapy was successfully completed to 14 days, resulting in the complete resolution of respiratory symptoms. The rapid resolution of symptoms and inflammatory markers within 72 h of initiating meropenem—following the failure of multiple prior outpatient antibiotic courses—strongly suggests that the clinical recovery was directly correlated with the targeted antimicrobial therapy. A follow-up HRCT performed one month after discharge demonstrated significant radiological regression of the acute inflammatory changes.

## 3. Discussion

A literature search of the PubMed database was conducted on January 15, 2026, for articles using the terms “*Pseudomonas fluorescens*” and “pneumonia”, supplemented by a snowball search of the reference lists of the retrieved articles (Supplementary File). To date, three cases of pneumonia have been reported in the literature in which *P. fluorescens* was isolated as the sole respiratory pathogen and considered the causative agent of the active infection (Table 2).

While *P. fluorescens* has traditionally been regarded as a non-pathogenic environmental organism, clinical reports have occasionally identified it as an opportunistic pathogen in certain clinical contexts. The *P. fluorescens* species complex is diverse, comprising multiple genomic groups with distinct ecological traits. Some studies suggest that certain lineages, such as Genomic Group III (*P. fluorescens sensu stricto*), may be more frequently associated with clinical isolates, potentially due to a greater capacity for growth at human body temperatures. However, the exact clinical significance of these genomic distinctions in daily practice remains to be fully elucidated [6].

Microbiome studies have provided insights into the presence of this organism in the respiratory tract. Researchers detected *P. fluorescens* sequences in bronchoalveolar lavage (BAL) samples analyzed by 16S rRNA sequencing, where a significant decrease in relative abundance upon removal of eukaryotic cells (*p* = 0.02) suggested a potential cell-associated nature [7]. While these molecular findings do not inherently prove pathogenicity, they indicate that the presence of *P. fluorescens* in the lower respiratory tract may be more frequent than conventionally recognized, suggesting it may persist as a part of the lung microbiota in certain contexts.

It has been hypothesized in the literature that the ability of *P. fluorescens* to persist within biofilms or intracellularly may render routine laboratory cultures inadequate for its detection, often leading to its potential misinterpretation as a colonizer in respiratory samples [4,8,9]. This proposed diagnostic challenge is particularly relevant to our case, as it provides a possible explanation for why the organism may be overlooked or dismissed unless a strong clinical–radiological correlation is established, especially following the failure of empirical therapies. Some studies suggest that the organism may be present in lower respiratory tract samples more frequently than conventionally accepted, though its detection is likely affected by diagnostic limitations and interpretive bias.

Previous reports also pointed to potential pathogenicity in lower respiratory tract infections, although the clinical interpretation of these early cases remains complex. For instance, *P. fluorescens* was isolated from a patient with an acute exacerbation of chronic bronchitis who developed resistance to imipenem [3]. However, as noted in the literature, the presence of potential confounders in such historical cases, including concomitant infections or extensive prior antibiotic use, can make the definitive attribution of pneumonia to *P. fluorescens* challenging. Additionally, the organism was identified in a patient participating in a study for community-acquired pneumonia, where the isolate persisted until alternative antibiotics were used [10]. While these studies did not primarily focus on *P. fluorescens* pneumonia, they demonstrate that the organism has been isolated from human respiratory tract infections, highlighting the long-standing challenge of distinguishing its pathogenic role from colonization.

To date, four culture-confirmed cases of *P. fluorescens* pneumonia have been described, including the present report. These cases typically involve older male patients where *P. fluorescens* was isolated as the sole potential pathogen, often after empirical treatment failure. The 1986 report by Thangkhiew involved a patient with a complex clinical course, including a concomitant abdominal infection and exposure to multiple antimicrobial agents; this complexity has led to scholarly debate regarding the definitive attribution of the pneumonia solely to *P. fluorescens* [11]. In contrast, more recent documentations have utilized advanced diagnostic techniques to establish a clearer causal link. Liu et al. described a 65-year-old male with structural lung disease where the diagnosis was definitively confirmed via transbronchial lung biopsy, providing direct histopathological evidence of the organism within the lung tissue [12]. Similarly, Ishii et al. reported a 71-year-old patient where pathogenicity was substantiated by sputum Gram staining that demonstrated the phagocytosis of Gram-negative rods by neutrophils—a critical finding that distinguishes active infection from simple surface colonization [13]. The consistency of these findings supports the interpretation that *P. fluorescens* may act as an opportunistic lower respiratory tract pathogen in susceptible hosts rather than merely a colonizer. Our case aligns with these documented reports but is further distinguished by the repeated isolation of the organism across multiple independent samples and a susceptibility profile limited to carbapenems, which accounts for the failure of prior broad-spectrum empirical therapies. Furthermore, the patient’s underlying structural lung disease and history of adenocarcinoma may have created a favorable environment for this opportunistic pathogen.

**Table 2:** Reported culture-confirmed cases of *Pseudomonas fluorescens* pneumonia.

Author (Year)	Age/Sex	Comorbidities	Diagnosis Method	Radiologic Features	Clinical Settings	Treatment	Clinical Outcome
Thangkhiew (1986) [11]	55/M	Miyastenia Gravis	Tracheal aspiration culture	CXR: bilateral consolidation	Postoperative pneumonia	Ceftazidime (metranidazole for abdominal infection)	Full Recovery
Liu (2021) [12]	67/M	Post Tuberculosis, Gastritis	Percutaneous lung biopsy	CT: bilateral nodular infiltration, unilateral pleural effusion	CAP	Ciprofloxacin	Full Recovery
Ishii (2024) [13]	80/M	COPD, DM, Rectal Cancer surgery	Sputum culture	CT: left lower lobe infiltration	HAP	Piperacillin/tazobactam	Full Recovery
Akgün (2026)	72/M	COPD, History of Prostate Cancer	Sputum culture (×2)	CT: bilateral reticulonodular infiltration	CAP	Meropenem	Full Recovery

CAP = community-acquired pneumonia; COPD = Chronic Obstructive Pulmonary Disease, DM = Diabetes Mellitus, HAP = hospital-acquired pneumonia; CXR = chest X-ray; CT = computed tomography.

This diagnostic dilemma is not unique to *P. fluorescens*; it is similar to the clinical challenges encountered with other opportunistic, non-fermenting Gram-negative bacilli, such as *Acinetobacter baumannii*, *Burkholderia cepacia*, and *Stenotrophomonas maltophilia* [14]. Consequently, the isolation of these opportunistic pathogens from respiratory secretions may represent colonization rather than active infection. However, they should be considered true respiratory pathogens when their isolation coincides with acute clinical deterioration, new or progressing radiological infiltrates, the failure of empirical therapies, and the rigorous exclusion of more common etiologies [15].

A notable limitation of this case report is the reliance on an automated phenotypic identification system (VITEK 2) rather than molecular confirmation (e.g., 16S rRNA sequencing), which is required to definitively differentiate *P. fluorescens* from closely related members of its species complex. While advanced molecular analyses represent the gold standard for microbiological characterization, they are often inaccessible in routine acute clinical care. Despite this limitation, we believe the repeated isolation of the organism, combined with the patient’s successful response to targeted therapy, provides valuable clinical observations regarding the management of this rare opportunistic pathogen. Moving forward, integrating molecular confirmation into routine diagnostic workflows in tertiary care centers will be essential to better distinguish these opportunistic organisms and refine targeted antimicrobial strategies.

## 4. Conclusions

While traditionally considered an environmental colonizer, *P. fluorescens* can emerge as a significant respiratory pathogen in susceptible patients. Distinguishing true infection from colonization requires integrating high-quality microbiological data with clinical–radiological correlation. While the number of documented cases is limited, current data indicate that the prognosis is generally favorable when timely and targeted antimicrobial treatment is applied. Continuous recognition and reporting of such cases is crucial for elucidating the clinical spectrum of *P. fluorescens* respiratory infections. Furthermore, genomic typing in future cases—particularly in centers with advanced molecular capabilities—could provide valuable insights into the strain distribution. Such studies might help determine whether specific groups, such as Group 3, contribute to human pathology, thereby addressing a significant knowledge gap in the current literature.

## Figures and Tables

**Figure 1:**
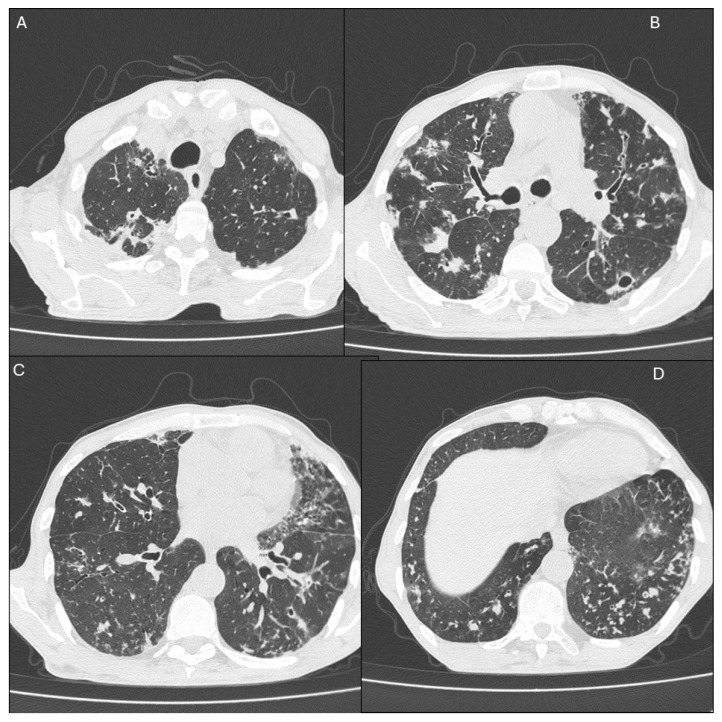
HRCT of the chest demonstrating bronchial wall thickening associated with underlying bronchiectasis, and tree-in-bud opacities compatible with active pulmonary infection.

**Table 1:** Antimicrobial Susceptibility Profile of the *P. fluorescens* Isolate.

Antimicrobial Agent	Minimum Inhibitory Concentration (MIC) (mg/L)	Interpretation
Meropenem	≤25	Susceptible
Amikacin	≤1	Susceptible
Imipenem	0.5	Susceptible dose-dependent (Intermediate)
Cefepime	≤0.12	Intermediate
Ciprofloxacin	≤0.06	Intermediate
Piperacillin/tazobactam	≤4	Intermediate
Ceftazidime/avibactam *		Resistant

* MIC value was not quantitatively reported; interpretation is based on the initial clinical microbiology report.

## Data Availability

The data supporting the findings of this study are available within the article and its Supplementary Materials.

## Supplementary Materials

The following supporting information can be downloaded at: https://www.mdpi.com/article/10.3390/microorganisms14061169/s1, Table S1. Non-clinical/Non-human studies excluded from clinical synthesis, Table S2. Non-English studies excluded from clinical synthesis, Table S3. Abstract-only studies excluded from clinical synthesis.

## Abbreviations

The following abbreviations are used in this manuscript:

COPD	Chronic Obstructive Pulmonary Disease
HRCT	High-resolution computed tomography
AFB	Acid-fast bacilli
CRP	C-reactive protein
NTM	Nontuberculosis mycobacteria
EUCAST	European Committee on Antimicrobial Susceptibility Testing
DM	Diabetes Mellitus
