# Supplementary material for: Pseudomonas fluorescens Pneumonia: A Case Report and Review of the Literature
Source: Microorganisms. 2026 May 22;14(6):1169. doi: 10.3390/microorganisms14061169 (PMC13304316; doi:10.3390/microorganisms14061169)
Supplement: Supplementary file 1 [file microorganisms-14-01169-s001.zip › microorganisms-4263319-supplementary.pdf]

Case Report

Pseudomonas fluorescens Pneumonia: A Case Report and Review of the Literature

Kadir Burak Akgün[1]

[1]Department of Pulmonology, Hatay Mustafa Kemal University, Hatay, Türkiye

Corresponding author: Kadir Burak Akgün, e-mail: kadirburakakgun@gmail.com, phone: +905448127828, ORCID: 0000-0002-3017-1025

Table S1. Non-clinical / Non-human studies excluded from clinical synthesis

Author	Year	Author	Year	Author	Year	Author	Year
Adegoke	1994	Herve	2024	Oleas-Paz	2019	Tsuji	1982
Anand	2010	Huws	2005	Pandya	2013	Veluchamy	2008
Arias	2003	Ibeyaima	2017	Papadopoulou	2008	Waso	2019
Arun	2015	Jabrane	2002	Polesky	2001	Waso-Reyneke	2022
Aurass	2013	Jackson	2001	Rasmussen	2000	Wróbel	2023
Barry	1990	Jones	2018	Rattö	2001	Yusuf	2012
Bellamy	1992	Kanaan	2024	Rouf	1968	Zaghloul	2025
Bharti	2022	Khan	2017 (May)	Ruiz	2013		
Birol	2021	Khan	2016	Sağdiç	2005		
Bisht	2022	Khan	2017 (Aug)	Saikia	2011		
Bonjar	2004	Kjaergaard	2000	Sankarganesh	2017		
Chazal	1995	Knichel	1982	Santos	2010		
Chung	1990	Kozlowski	1988	Sarjeant	2005		
Chung	2007	Lillie	2024	Schnider	1995		
Csakvari	2021	Lu	1998	Schöbel	2023		
Delmon	2023	Macrae	1972	Seeley	1979		
Denter	1994	Makhlof	2024	Siddique	2007		
Dias	2018	Matsushita	1982	Soda	2008		
Dias	2019	Mengistu	2022	Spindler-Raffel	2017		
Eutenuer	1985	Mergeay	1978	Stalon	1987		
Faulde	2012	Mosharaf	2018	Stewart	2012		
Filali	2000	MubarakAli	2015	Stoodley	1997		
Gaeta	2017	Nakata	2000 (Mar)	Stoodley	1994		
Ginde	2025	Nakata	2000 (Oct)	Stoodley	1998		
Gurijala	1988	Neu	1987	Stoodley	1999		
Haque	2021 (Jan)	Norberg	2011	Syed	2016 (Jul–Sep)		
Haque	2021 (Mar)	Nowak	2021	Syed	2016 (Dec)		
Harshiny	2015	Oie	2012	Tachikawa	2005		
Henis	1990	Okla	2012	Thakur	1995		

Table S2. Non-English studies excluded from clinical synthesis

Author	Year	Language	Author	Year	Language
Amemiya	1994	Japanese	Olszewski	2008	Polish
Bompard	1988	French	Popov	2011	Russian
Hinz	1992	German	Uematsu	2020	Japanese
Igari	2002	Japanese	Yamamoto	1991	Japanese
Igari	2003	Japanese	Ye	2012	Chinese
Laguna-del Estal	2010	Spanish			

Table S3. Abstract-only studies excluded from clinical synthesis

Author	Year	Mini-notes
Belobraydic	1986	General clinical context, no patient details
Hessen	1987	Osteomyelitis case report
Qadri	1986	General clinical, 2 patient isolates
Terleckyj	1983	No abstract or full text available
Vollmar	2014	*B. pseudomallei* osteomyelitis; blood culture grew *P. fluorescens* 6 years earlier during pneumonia episode
